# Gene expression and DNA methylation altering lead to the high oil content in wild allotetraploid peanut (*A. monticola*)

**DOI:** 10.3389/fpls.2022.1065267

**Published:** 2022-12-16

**Authors:** Nian Liu, Bei Wu, Manish K. Pandey, Li Huang, Huaiyong Luo, Yuning Chen, Xiaojing Zhou, Weigang Chen, Dongxin Huai, Bolun Yu, Hao Chen, Jianbin Guo, Yong Lei, Boshou Liao, Rajeev K. Varshney, Huifang Jiang

**Affiliations:** ^1^ Key Laboratory of Biology and Genetic Improvement of Oil Crops, Ministry of Agriculture and Rural Affairs, Oil Crops Research Institute of the Chinese Academy of Agricultural Sciences, Wuhan, China; ^2^ Center of Excellence in Genomics and Systems Biology (CEGSB), International Crops Research Institute for the Semi-Arid Tropics (ICRISAT), Hyderabad, India; ^3^ Institute of Crop Sciences, Fujian Academy of Agricultural Sciences, Fuzhou, China

**Keywords:** *Arachis monticola*, *Arachis hypogaea*, oil biosynthesis, expression profiling, DNA methylation, functional genomics

## Abstract

**Introduction:**

The wild allotetraploid peanut *Arachis monticola* contains a higher oil content than the cultivated allotetraploid *Arachis hypogaea*. Besides the fact that increasing oil content is the most important peanut breeding objective, a proper understanding of its molecular mechanism controlling oil accumulation is still lacking.

**Methods:**

We investigated this aspect by performing comparative transcriptomics from developing seeds between three wild and five cultivated peanut varieties.

**Results:**

The analyses not only showed species-specific grouping transcriptional profiles but also detected two gene clusters with divergent expression patterns between two species enriched in lipid metabolism. Further analysis revealed that expression alteration of lipid metabolic genes with co-expressed transcription factors in wild peanut led to enhanced activity of oil biogenesis and retarded the rate of lipid degradation. In addition, bisulfite sequencing was conducted to characterize the variation of DNA methylation between wild allotetraploid (245, WH 10025) and cultivated allotetraploid (Z16, Zhh 7720) genotypes. CG and CHG context methylation was found to antagonistically correlate with gene expression during seed development. Differentially methylated region analysis and transgenic assay further illustrated that variations of DNA methylation between wild and cultivated peanuts could affect the oil content *via* altering the expression of peroxisomal acyl transporter protein (*Araip.H6S1B*).

**Discussion:**

From the results, we deduced that DNA methylation may negatively regulate lipid metabolic genes and transcription factors to subtly affect oil accumulation divergence between wild and cultivated peanuts. Our work provided the first glimpse on the regulatory mechanism of gene expression altering for oil accumulation in wild peanut and gene resources for future breeding applications.

## Introduction

Cultivated peanut (*Arachis hypogaea* L.) is one of the important oil crops, which is widely grown in more than 100 countries. The annual production is 48.76 Mt, with 29.60 Mha of global planting area for cultivated peanut (FAOSTAT, 2019, https://www.fao.org/faostat/en/#data/). It was domesticated from a wild relative, *Arachis monticola*, which harbors a high oil content and resistance to several biotic stresses (Bertioli et al., 2011; Huang et al., 2012; Moretzsohn et al., 2013; Yin et al., 2020). Since cultivated peanut is one of the major sources of edible oil in the world, enhancing oil content is the second most vital objective after yield in peanut breeding. There are examples in several crops that novel genes from wild counterparts were successfully introgressed into cultivated accessions for crop improvement (Hufford et al., 2012; Sang and Ge, 2013; Qi et al., 2014; Tian et al., 2019). The wild counterpart (*A. monticola*) not having any reproductive barrier with domesticated peanut (*A. hypogaea*) could provide favorable alleles for high oil accumulation. Developing a better understanding of the molecular mechanism and genomic control of high lipid accumulation in wild peanut would facilitate a significant increase in oil content in newly developed peanut varieties.

Lipids can be classified into fatty acids (FAs), galactolipids, phospholipids, sphingolipid, and acylglycerol (Manan et al., 2017). Tri-acylglycerol (TAG) is the major form of lipid in seed oil that provides calories and essential nutrients to the human body. In the model plant *Arabidopsis*, significant progress has been made in understanding lipid biosynthesis, transport, and degradation. Many genes encoding enzymes involved in lipid metabolism have been characterized, and several transcription families such as B3, NFY-B, AP2/EREBP, and bZIP have been reported to regulate these structural genes to control seed oil accumulation (Beisson, 2003; Li-Beisson, 2013; Manan et al., 2017). Only a couple of studies on expression variation underlying oil accumulation of peanut seed are available for domesticated peanut, *A. hypogaea* (Wang et al., 2018; Zhang et al., 2021). In the case of the wild tetraploid, *A. monticola*, a study very recently has been reported on the alteration of gene expression by structural variations affecting pod size (Yin et al., 2020) and not oil accumulation. Ample literature generated in the past provided convincing evidence related to the alteration of gene transcription contributing to phenotypic variations between domesticated crops and their wild counterparts (Koenig et al., 2013; Ichihashi et al., 2014; Yoo and Wendel, 2014; Lu et al., 2016). The last decade has witnessed monumental progress in terms of genomic resources including reference genomes, gene expression atlas, and genotyping assays in peanut to accelerate the genomics and breeding applications in peanut (Pandey et al., 2020). The availability of reference genomes for wild and cultivated tetraploid provides a unique opportunity to explore comparative structural and functional genomics to generate more information to further expand our understanding of the genomic and regulatory mechanisms of enhanced oil accumulation in *A. monticola*.

In addition to gene transcription, recent studies have also revealed the association between the alteration of DNA methylation and phenotypic variation in crops (Shen et al., 2018; Xu et al., 2019). In plants, DNA methylation occurs in the three sequence contexts of cytosine, namely, CG, CHG, and CHH (H represents A, T, or C) (Zhang et al., 2006; Zhang et al., 2018). In heterochromatin or euchromatic chromosome arms, DNA methylation plays a role in the control of transposon silencing and chromosome interaction (Zhang et al., 2006; Cokus et al., 2008). DNA methylation in gene promoters or bodies usually represses transcription but might increase transcription in some cases (Zhang et al., 2006; Wang et al., 2015; Bewick and Schmitz, 2017; Lang et al., 2017; Huang et al., 2019). The DNA methylation has been widely reported to play important roles in vegetable growth, fruit ripening, seed development, and response to biotic and abiotic stress (Zhong et al., 2013; Baubec et al., 2014; Yong-Villalobos et al., 2015; Hewezi et al., 2017; Narsai et al., 2017; Huang et al., 2019; Rajkumar et al., 2020). However, the role of DNA methylation on the regulation of protein-coding genes involved in oil accumulation is largely unknown including in peanut. Integrated analysis of methylome and transcriptome data would investigate the alteration of DNA methylation and its association with genome-wide gene expression. Therefore, bisulfite sequencing (bisulfite-seq) and RNA sequencing (RNA-seq) technologies have been performed to reveal methylation regulation of genes involved in abiotic resistance, fruit development, and reproductive growth (Yong-Villalobos et al., 2015; Huang et al., 2019; Gutschker et al., 2022; You et al., 2022). These studies would expand our view of DNA methylation on the regulation of gene transcription.

In the present study, we conducted comparative transcriptome profiling of developing seeds from multiple accessions of wild and cultivated peanuts using RNA-seq. Two developmental stages in seed were studied, R5 and R8, representing stages of lipid initial accumulation and lipid rapid increase, respectively. In addition, bisulfite-seq was performed to investigate the variation of DNA methylation between wild accession (245, WH 10025) and cultivated accession (Z16, Zhh 7720). The objective of this study is to reveal a regulatory mechanism of gene expression altering for enhanced oil accumulation in peanut.

## Materials and methods

### Plant material

Three accessions, 171 (WH 4335), 172 (WH 4334), and 245 (WH 10025), were collected to represent wild peanuts (*A. monticola*). Five accessions, 003 (Zhh 0225), 145 (Zhh 0888), 492 (Zhh 0003), 502 (Zhh 0602), and Z16 (Zhh 7720), were used to represent cultivated peanuts (*A. hypogaea*). Among them, 003, 145, 492, and 502 belong to four mainly agronomic types (*vulgaris*, *hypogaea*, *fastigiata*, and *hirsuta*, respectively) in cultivated peanut. Z16 is a modern cultivar with mixed parentage. Wild and cultivated peanuts were planted in the experimental nursery of Oil Crops Research Institute of Chinese Academy of Agricultural Sciences, Wuhan. Nursery management followed standard agricultural practices. Three biological replications were grown for each accession. Developing seeds from eight accessions were collected at previously characterized stages (Pattee et al., 1974): lipid initial accumulation stage (R5) in which developing seeds are smaller and lipid rapid increase stage (R8) in which developing seeds are bigger. The collected samples were immediately frozen in liquid nitrogen and stored at -70°C for RNA and DNA isolation. The oil content (%) of mature seeds from each accession was measured as described previously (Liu et al., 2020).

### Processing of RNA sequencing data

Total RNA was extracted using an RNAprep pure plant kit (Tiangen, Beijing, China) according to the manufacturer’s instructions. Forty-eight libraries from the developing seeds of wild and cultivated peanuts were constructed and sequenced using a HiSeq XTen platform in Beijing Genomics Institute (BGI; https://www.genomics.cn/). After obtaining raw data, we used software SOAPnuke to perform quality filtering and read trimming (https://github.com/BGI-flexlab/SOAPnuke). The clean RNA-seq reads were mapped to the reference genome using HISAT2 software (Gao et al., 2016). The parameters were set as follows: –phred64 –sensitive –no-discordant –no-mixed –mp 6,2 -X 1000. The reference genome consisted of two diploid ancestors’ genomes of *Arachis ipaensis* (V14167) and *Arachis duranensis* (V14167) (Bertioli et al., 2016). The RSEM package was used to calculate and normalize the gene expression level as fragments per kilobase of transcript per million mapped reads (FPKM) (Koenig et al., 2013). Differentially expressed genes (DEGs) with FPKM ≥1 in at least one sample (fold change ≥2 and adjusted *P-*value ≤0.001) were identified using DEGseq package (Lin et al., 2012).

### Gene clustering, functional annotation, and weighted gene co-expression network analysis

K-means clustering was used to visualize genes exhibiting a similar expression pattern, and it was performed on normalized FPKM values using MeV software (Saeed et al., 2003). The distance metric for K-means clustering was set as Euclidean distance. Annotation analyses were performed by BLASTing public protein databases, including Nr (http://www.ncbi.nlm.nih.gov), Gene Ontology (GO) (http://www.geneontology.org), and Kyoto Encyclopedia of Genes and Genomes (http://www.genome.jp/kegg). GO annotation of all of the genes in the two diploid ancestors’ genomes was downloaded from the website AgriGO (http://bioinfo.cau.edu.cn/agriGO/index.php) and was set as background reference to identify and show overrepresented GO terms using software TBtools (Chen et al., 2020). The R package [weighted gene co-expression network analysis (WGCNA)] was used to build weighted gene co-expression networks (Langfelder and Horvath, 2008). Network construction was performed using the blockwiseModules function with default parameters. The topological overlap matrix (TOM) was calculated to measure the strength of a co-expression relationship, i.e., connectivity between any two genes with respect to all other genes in the network. The expressed genes with FPKM ≥1 in at least one sample were selected to perform K-means clustering and WGCNA.

### Whole-genome bisulfite sequencing

Genome DNA from each sample was extracted using a DNeasy Plant Maxi Kit (Qiagen, Germany). Then, DNA was fragmented to a mean size of 250 bp through Bioruptor (Diagenode, Belgium). Adapters were ligated to the fragment DNA and treated with sodium bisulfite using EZ DNA Methylation-Gold Kit (Zymo, USA). Sequencing was performed using Illumina HiSeq platform in paired-end mode at BGI (https://www.genomics.cn/). Three biological replications were sequenced for each stage of developing seeds in both wild and cultivated peanuts.

### Read alignment and methylcytosine identification

The raw reads from each library were processed to remove low-quality reads, adaptor sequences, and contamination using software Trimmomatic (Bolger et al., 2014). Bisulfite sequence mapping program (BSMAP) was conducted to map clean reads to the reference genome with parameters (-u -v 8 -z 33 -p 4 -n 0 -w 20 -s 16 -f 10 -L 150), and only the reads mapped at unique positions were retained (Xi and Li, 2009). The binomial test was performed for each cytosine base to identify true methylcytosine (mC). Only the cytosines covered by at least four reads in all compared tissues were considered for further analysis. Cytosine sites with *P*-value <0.0001 were defined as methylated cytosine sites. The methylation level at each mC site was determined by the percentage of reads giving a methylation call to all of the reads aligned at the same site.

### Identification of differentially methylated regions

Putative differentially methylated regions (DMRs) were identified using windows that contained at least five CG (CHG or CHH) sites with different methylation levels (cutoff value >0.1 between two compared samples) and Fisher test *P*-value ≤0.05. In addition, two nearby DMRs would be considered interdependent and joined into one continuous DMR if the genomic region from the start of an upstream DMR to the end of a downstream DMR also had different methylation levels (cutoff value >0.1 between two compared samples) with a *P*-value ≤0.05. Otherwise, the two DMRs were viewed as independent. When the genic region (2 kb upstream or body) was overlapped with DMR, the gene was defined as a DMR-associated gene. The fold enrichment of DEGs in DMR-associated genes was calculated as (DMR-associated DEGs/Total DEGs)/(DMR-associated genes/Total genes), and *P*-value significance was generated using the hypergeometric test.

### Plant transformation

The full-length open reading frame (ORF) of *CTS* (*Araip.H6S1B*) was cloned and subsequently recombined into pBWA(V)HS plasmid (from Wuhan BioRun Biosciences Co., Ltd., China) to generate *35S:CTS* construct. *Arabidopsis* plant transformation was performed by the floral dip method (Zhang et al., 2006). Peanut cotyledons from germinating seeds were used as explant for hairy root transformation following a previous report (Yuan et al., 2019).

### RNA isolation and qRT-PCR

Total RNA was isolated using plant RNA Extract Kit (Tiangen Biotech, China). The first-strand cDNA was synthesized following the instruction of Revert Aid First Strand cDNA Synthesis Kit (Thermo Fisher, USA). qRT-PCR was performed using a CFX Connect Real-time system (Bio-Rad, USA) with ChamQ Universal SYBR qPCR Mix (Vazyme, China). The relative expression levels were calculated using the 2^-ΔCT^ method. *GAPDH* (Morgante et al., 2011) and *AtACTIN7* (AT5G09810) were chosen as reference genes to normalize the relative expression level of *CTS* (*Araip.H6S1B*) in peanut and *Arabidopsis*, respectively.

### Fat red 7B staining

Fat red staining was performed by incubating *Arabidopsis* seedlings and peanut hairy roots in 0.1% (w/v) Fat red 7B solution for 3 h at room temperature. Samples were then rinsed with 70% ethanol to remove chlorophyll. The samples were quickly photographed using a stereomicroscope (Olympus SZX16, Japan).

## Results

### Evaluation of RNA sequencing data

**Figure 1:**
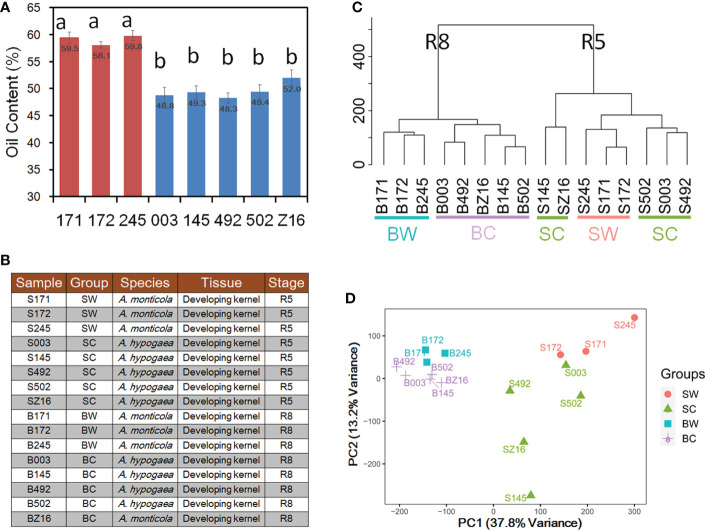
Analysis of wild and cultivated peanuts at two seed developmental stages. **(A)** Seed oil content of three wild and five cultivated accessions. **(B)** Illustration of 16 samples for RNA sequencing (RNA-seq). Different letters indicated statistically significant differences in oil content between accessions according to Tukey’s range test at the 0.05 level. **(C)** Hierarchical clustering between 16 samples. **(D)** Principal component analysis (PCA) of gene expression profiles in 16 samples. SW and SC indicated seeds of wild and cultivated peanuts at the R5 stage, respectively. BW and BC denoted seeds of wild and cultivated peanuts at the R8 stage, respectively.

To analyze phenotypic variations in seed oil accumulation between wild tetraploid and cultivated peanuts, we measured oil content in three accessions of *A. monticola* and five accessions of *A. hypogaea*. The oil content of mature seed in three wild accessions, namely, 171 (WH 4335), 172 (WH 4334), and 245 (WH 10025), ranged from 58.1% to 59.8% and that of five cultivated peanuts, namely, 003 (Zhh 0225), 145 (Zhh 0888), 492 (Zhh 0003), 502 (Zhh 0602), and Z16 (Zhh 7720), ranged from 48.3% to 52.0% (**Figure 1A**). Multiple comparison analyses indicated that wild tetraploid species have significantly higher oil content than cultivated peanuts. To explore the reason for oil accumulation difference during peanut domestication, developing seeds at two stages (R5 and R8) were collected to generate RNA-seq datasets for both species (**Figure 1B**). Sixteen samples with three biological replications were used to construct RNA-seq libraries followed by generation of 44.1–45.4 million clean reads per library (**Table S1**). The union set of genomes of diploid ancestors *A. ipaensis* and *A. duranensis* (Bertioli et al., 2016) was used as a reference in this study. Averages of 83.72% and 83.59% of clean reads were mapped on the reference genome for wild and cultivated peanuts, respectively (**Table S1**). This similar mapping rate indicated that the reference can be used to quantify the gene expression level in both wild and cultivated species. The number of genes among different libraries ranged from 32,148 to 45,226 genes with the expression of a total of 56,236 genes among 16 samples (**Tables S1**, **S2**). Hierarchical clustering analysis was performed to cluster the samples according to developmental stages (**Figure 1C**). In the stage R8 group, the samples could be further divided into wild (BW) or cultivated (BC) subgroups. However, the samples in the R5 group could not be clearly distinguished between wild and cultivated subgroups. The first two principal component analysis (PCA) components (**Figure 1D**) of transcriptional profiles explained 37.8% (PC1) and 13.2% (PC2) of sample-to-sample variance. Similar to hierarchical clustering analysis, the PCA also grouped samples according to developmental stages (PC1) prior to species (PC2).

### Differentiation in gene expression between wild and cultivated peanuts

**Figure 2:**
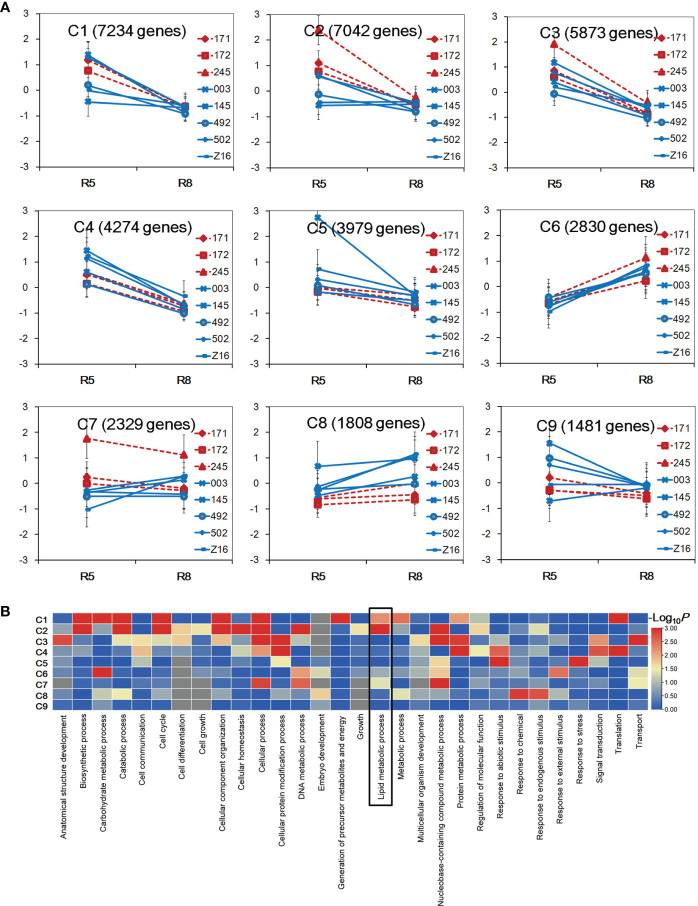
Gene expression pattern between wild and cultivated peanuts. **(A)** Identification of gene clusters in 16 samples. Nine gene clusters (C1–C9) were identified using K-means clustering. The x-axis represented the stage of developing seed. The y-axis represented log2 FPKM derived from RNA sequencing (RNA-seq) data for each sample FPKM fragments per kilobase of transcript per million mapped reads. **(B)** Heatmap of enrichment of biological process category among nine clusters. The color was indicated –log10 (*P*-value). The lipid metabolism process was boxed in the heatmap.

To explore the divergence of gene expression patterns between two species, transcriptional profiles of 16 samples were used to perform K-mean clustering analysis. A total of 36,850 genes with FPKM ≥1 in at least one sample were selected to be grouped into nine clusters designated as C1–C9 (**Figure 2A**). Five clusters (C1–C5) showed a decreasing tendency from the R5 to R8 stage in both peanut species. Conversely, the C6 and C8 clusters showed an increasing trend across accessions from the R5 to R8 stage. For the C7 cluster, wild accessions (171, 172, and 245) showed a decreasing trend, while cultivated accessions showed an increasing pattern or no obvious change during seed developmental stages. In the C2 cluster, the gene expression level was higher in wild accessions at the R5 stage. In the C8 cluster, the gene expression level was in general higher in cultivated accessions at both stages. GO category analysis was performed to identify overrepresented GO terms of the nine clusters (**Figure 2B**, **Table S3**). A total of 30, 14, and 22 GO terms were enriched in biological process, cellular component, and molecular function, respectively. Each cluster had differently enriched GO terms, and the number of overrepresented GO terms ranged from 3 (C9) to 35 (C2). The GO term of lipid metabolism process was found overrepresented in clusters C1, C2, and C7 (*P* < 0.05). Interestingly, the gene expression level was different between wild and cultivated peanuts in C2 and C7 clusters, indicating that the transcriptional profile of lipid metabolism may be divergent between the two species.

**Figure 3:**
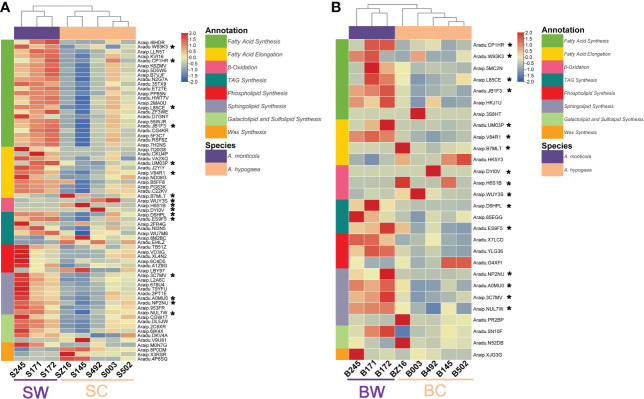
Heatmaps of lipid metabolism-related DEGs between wild and cultivated peanuts. Panels **(A)** and **(B)** represented the R5 and R8 stages, respectively. * denoted the differentially expressed genes identified at both stages. SW and SC indicated seeds of wild and cultivated peanuts at the R5 stage, respectively. BW and BC denoted seeds of wild and cultivated peanuts at the R8 stage, respectively.

To further dissect the expression difference of lipid metabolism, a comparative transcriptomic analysis was performed to identify DEGs between wild and cultivated peanuts. There were 5,647 and 3,184 DEGs at the R5 (SW vs. SC) and R8 (BW vs. BC) stages, respectively (**Figure S1**; **Tables S4**, **S5**). A total of 1,578 DEGs were detected at both R5 and R8 stages. The number of upregulated and downregulated DEGs was overall equivalent at both R5 and R8 stages (**Figure S1**). Heatmaps of DEGs involved in FA synthesis, FA elongation, TAG synthesis, phospholipid synthesis, sphingolipid synthesis, galactolipid and sulfolipid synthesis, wax synthesis, and β-oxidation were profiled to represent a transcriptional change in lipid metabolism (**Figure 3**). According to the expression pattern of DEGs involved in lipid metabolism, samples in both heatmaps (R5 and R8 stages) could be divided into two groups (wild and cultivated). At the R5 stage, most lipid metabolism-related pathways, such as FA synthesis, FA elongation, TAG synthesis, phospholipid synthesis, sphingolipid synthesis, and galactolipid and sulfolipid synthesis were dramatically upregulated in wild species (**Figure 3A**). Most downregulated DEGs at the R5 stage were mainly distributed on β-oxidation and wax synthesis pathways. Compared with the R5 stage, the portion of upregulated DEGs in wild species was generally lower at the R8 stage (**Figure 3B**). However, DEGs in FA synthesis, TAG synthesis, phospholipid synthesis, and sphingolipid synthesis were mainly upregulated in wild peanut accessions at the R8 stage. Conversely, all of the DEGs in β-oxidation were downregulated at the R8 stage. There were 16 lipid metabolic DEGs simultaneously identified at both R5 and R8 stages belonging to FA synthesis (5), FA elongation (3), TAG synthesis (2), sphingolipid synthesis (4), and β-oxidation (3) pathways. According to the results of the clustering analysis, 81 lipid metabolism-related DEGs were grouped into eight clusters. About 44% of lipid metabolism-related DEGs (36) belonged to C2 clusters in which the expression pattern was divergent between wild and cultivated peanuts. Another two “divergent” clusters (C7 and C8) harbored six and five lipid metabolism-related DEGs (**Tables S4**, **S5**).

### Construction of the co-expression network

**Figure 4:**
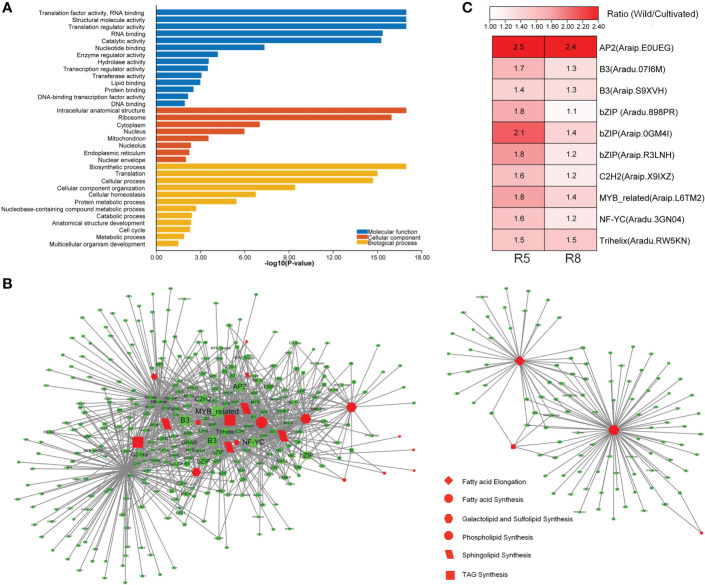
Identification of co-expression genes with DEGs involved in lipid metabolism. **(A)** GO enrichment analysis of co-expression genes. GO terms with *P*-value <0.05 were listed in the y-axis. **(B)** Network of co-expression TFs with lipid metabolic DEGs. **(C)** Differential expression of top 10 co-expression TFs between wild and cultivated peanuts. Ratio (Wild/Cultivated) represented fold change of FPKM values between wild and cultivated peanut. FPKM fragments per kilobase of transcript per million mapped reads.

To further explore genes with high connectivity to lipid metabolism-related DEGs, WGCNA (Langfelder and Horvath, 2008) was performed to construct a TOM of expression similarity between genes (**Table S6**). Genes co-expressed with lipid metabolism-related DEGs (expression similarity ≥0.2) were selected to perform GO enrichment analysis. They were enriched in 14 GO terms for molecular function category, including transcription regulator activity, DNA-binding transcription factor (TF) activity, and DNA binding (**Figure 4A**). It was suggested that TFs may play a role in the co-expression network. Many TFs were differentially expressed between wild and cultivated peanuts (**Figure S3**). In specific TF families, such as AP2, B3, and bZIP, the family members were predominantly upregulated in wild peanuts at both R5 and R8 stages. A total of 391 TFs belong to 47 families that were co-expressed with DEGs involved in lipid synthesis pathways, such as FA synthesis, FA elongation, TAG synthesis, phospholipid synthesis, sphingolipid synthesis, and galactolipid and sulfolipid synthesis (**Figure 4B**). According to the interaction number, the top 10 notes in the co-expressed TFs (**Table S6**) were AP2 (*Araip.E0UEG*), B3 (*Aradu.07I6M*), B3 (*Araip.S9XVH*), bZIP (*Aradu.898PR*), bZIP (*Araip.0GM4I*), bZIP (*Araip.R3LNH*), C2H2 (*Araip.X9IXZ*), MYB-related (*Araip.L6TM2*), NF-YC (*Aradu.3GN04*), and Trihelix (*Aradu.RW5KN*). Fold change (wild/cultivated) of the TFs ranged from 1.4 to 2.5 at the R5 stage with a mean value of 1.8, while the values ranged from 1.1 to 2.4 at the R8 stage with a mean value of 1.4 (**Figure 4C**). Interestingly, six of the top 10 co-expressed TFs, i.e., AP2 (*Araip.E0UEG*), B3 (*Aradu.07I6M*), bZIP (*Aradu.898PR, Araip.0GM4I, Araip.R3LNH*), and Trihelix (*Aradu.RW5KN*), were divided into the C2 clusters (**Table S6**). There was a quarter of co-expressed TFs (101) belonging to the C2 clusters in which the gene expression pattern was divergent between wild and cultivated peanuts. Among the co-expressed TFs in the C2 “divergent” clusters, 43 members including AP2, bZIP, ARF, C3H, C2H2, and HD-ZIP were significantly upregulated in wild peanuts, especially at the R5 stage. Together with co-expressed TFs, ~44% of lipid metabolism-related DEGs were also categorized into the C2 clusters, suggesting that there was a divergent TF module to regulate the expression of lipid metabolism-related genes between wild and cultivated peanuts.

### Influence of DNA methylation on gene expression

**Figure 5:**
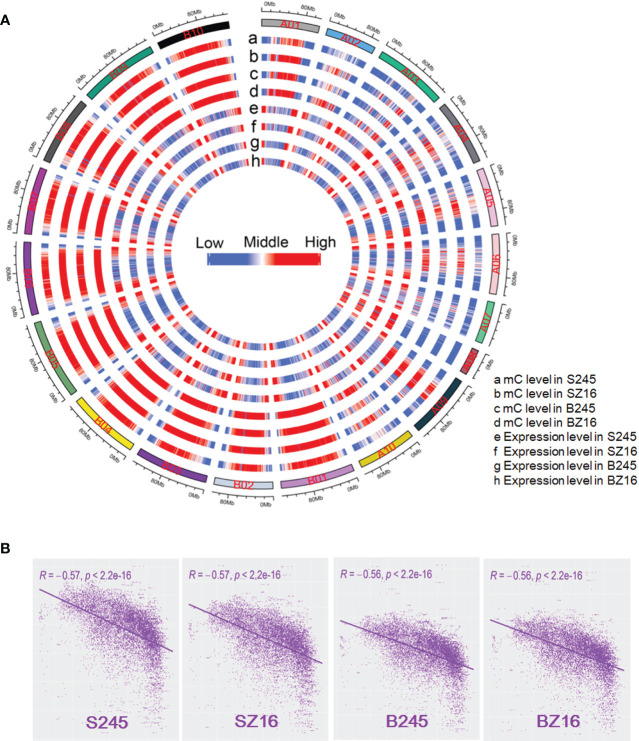
Genome-wide correlation between DNA methylation and gene expression. **(A)** A circle plot showed the overall DNA methylation level and gene expression level (log10 FPKM) in the reference chromosomes. FPKM fragments per kilobase of transcript per million mapped reads. mC indicated methylated cytosine. The data for each chromosome were analyzed in 1 Mb windows sliding 200 kb. **(B)** Analysis of correlation coefficient between DNA methylation and gene expression. S245 and SZ16 indicated seeds of 245 and Z16 at the R5 stage, respectively. B245 and BZ16 indicated seeds of 245 and Z16 at the R8 stage, respectively.

The available literature in multiple crops proved that DNA methylation could help regulate gene expression (Wang et al., 2015; Huang et al., 2019; Rajkumar et al., 2020; Wang et al., 2016; Xing et al., 2015). To reveal the relationship between DNA methylation and gene expression in peanut, wild peanut (245) with the highest oil content among the eight accessions and elite cultivar (Z16) with a relatively low oil content were selected to perform bisulfite-seq for developing seeds at the R5 and R8 stages. Four samples (S245, SZ16, B245, BZ16) representing seeds at the R5 (S) and R8 (B) stages for 245 and Z16 were sequenced with three biological replicates. About 480 M clean reads were generated for each sample (**Table S7**), and approximately 76% of the clean reads were uniquely mapped to the reference genomes covering >87% of the genomic cytosine positions. Each methylome had >16-fold average depth per strand. Methylcytosines were identified in CG, CHG, and CHH contexts across samples. Compared with the average methylation of the CHH context (11.2%–22.3%), the level was much higher in CG (81.7%–86.0%) and CHG (73.2%–78.2%) contexts (**Figure S3A**). The fraction of mC was 20.1%–25.2% in CG, 25.6%–31.8% in CHG, and 43.0%–54.4% in CHH. The overall methylation levels in the four samples were similar (29.3%–34.7%) (**Figure 5A**, **Figure S3A**). The distribution of mC showed much lower methylation in the terminal chromosomes in contrast to a much higher gene expression in the terminal chromosomes (**Figure 5A**). The genome-wide correlation coefficient between overall DNA methylation and gene expression in the four samples was ~-0.56 (*P* < 2.2 e-16), indicating a significantly antagonistic correlation (**Figure 5B**).

**Figure 6:**
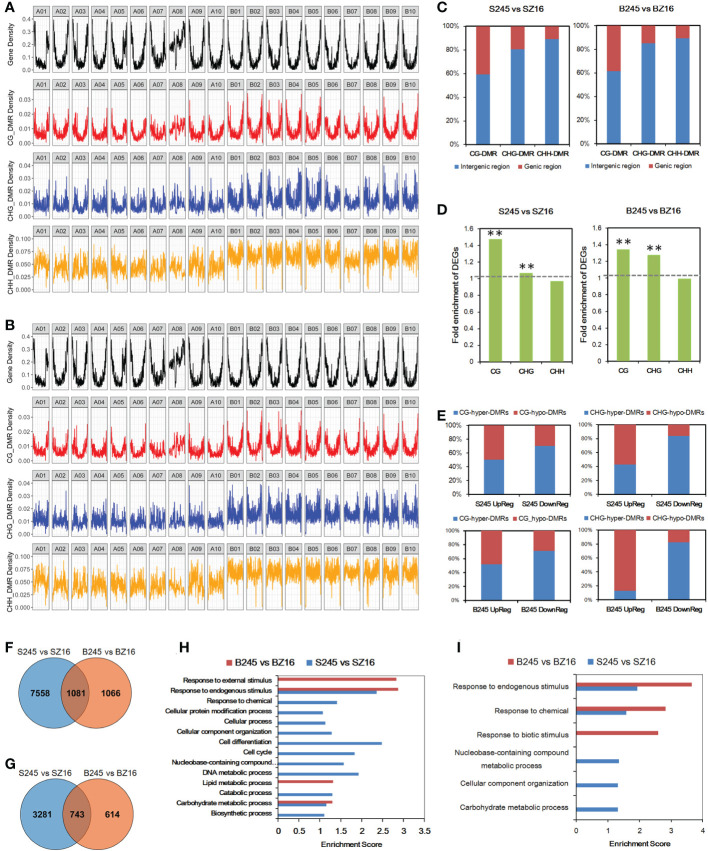
Influence of DMR on differential gene expression. **(A, B)** Genome-wide distribution of protein-coding genes and DMRs (CG, CHG, and CHH) between wild and cultivated seeds at the R5 stage **(A)** and R8 stage **(B)**. **(C)** Percentage of DMRs in the genic region and intergenic region. **(D)** Fold enrichment of DEGs in those overlapping with DMRs in the gene body with 2 kb upstream. The hypergeometric test was used to infer statistical significance (** *P* < 0.01). **(E)** The ratio of (hyper/hypo) DMRs in CG or CHG contexts overlapped with DEGs (up/down) between seeds of 245 and Z16 at the R5 and R8 stages. **(F)** Venn diagram of CG-DMR-associated DEGs at the R5 and R8 stages. **(G)** Venn diagram of CHG-DMR-associated DEGs between 245 and Z16 at the R5 and R8 stages. **(H)** GO enrichment analysis of CG-DMR-associated DEGs between 245 and Z16 at the R5 and R8 stages. GO terms with *P*-value <0.05 were listed in the y-axis. **(I)** GO enrichment analysis of CHG-DMR-associated DEGs between 245 and Z16 at the R5 and R8 stages. GO terms with *P*-value <0.05 were listed in the y-axis. S245 and SZ16 denoted seeds of 245 and Z16 at the R5 stage, respectively. B245 and BZ16 denoted seeds of 245 and Z16 at the R8 stage, respectively. DMR indicated differentially methylated regions.

To further characterize epigenetic variations between 245 (wild) and Z16 (cultivated), DMRs of CG, CHG, and CHH contexts were detected at the R5 and R8 stages (**Table S8**). The chromosome-wide view of DMR distribution indicated CG-DMR being the most likely to be enriched in gene-rich regions (**Figures 6A, B**). Approximately 40% of CG-DMRs were located in the genic region (gene body+2k upstream); the portions were reduced to ~15% and ~10% for CHG-DMRs and CHH-DMRs, respectively (**Figure 6C**). Conjoint analysis of the expression profile and DMRs indicated that CG-DMRs and CHG-DMRs were significantly enriched in the genic region (gene body+2k upstream) of DEGs between 245 and Z16 (**Figure 6D**). It is hinted that CG-DMR and CHG-DMR, not CHH-DMR, may play a role in the change of gene expression. Among DEG-overlapped CG-DMRs, the portion of hyper-DMRs in S245-upregulated DEGs was 50.3% and 51.9% at the R5 and R8 stages, respectively, and that in S245-downregulated DEGs could increase to 70.5% and 71.1% at the R5 and R8 stages, respectively (**Figure 6E**). Similarly, the portion of hyper-DMRs was much higher in S245-downregulated DEGs (83.7% and 82.4% at R5 and R8, respectively) than in S245-upregulated DEGs (42.7% and 12.5% at R5 and R8, respectively) for DEG-overlapped CHG-DMRs. The results suggested that the dynamic change of methylated CG (mCG) and methylated CHG (mCHG) on a genic scale correlated negatively with the difference in gene abundance between 245 and Z16.

### Role of DNA methylation in the divergence of lipid metabolism

The CG-DMRs between 245 and Z16 were overlapped with 8,639 and 2,147 DEGs at the R5 and R8 stages, respectively (**Figure 6F**). For CHG-DMRs, there were 4,024 and 1,357 CHG-DMR-associated DEGs at the R5 and R8 stages, respectively (**Figure 6G**). In general, more than 50% and 25% of DEGs between 245 and Z16 may be associated with CG-DMRs and CHG-DMRs, respectively. GO enrichment analysis showed that CG-DMR-associated DEGs were enriched in the carbohydrate metabolic process and response to endogenous stimulus at both stages (**Figure 6H**). The CHG-DMR-associated DEGs were enriched in response to chemical and response to endogenous stimulus at both stages (**Figure 6I**). Interestingly, lipid metabolism was one of the enriched terms at the R8 stage for CG-DMR-associated DEGs.

**Figure 7:**
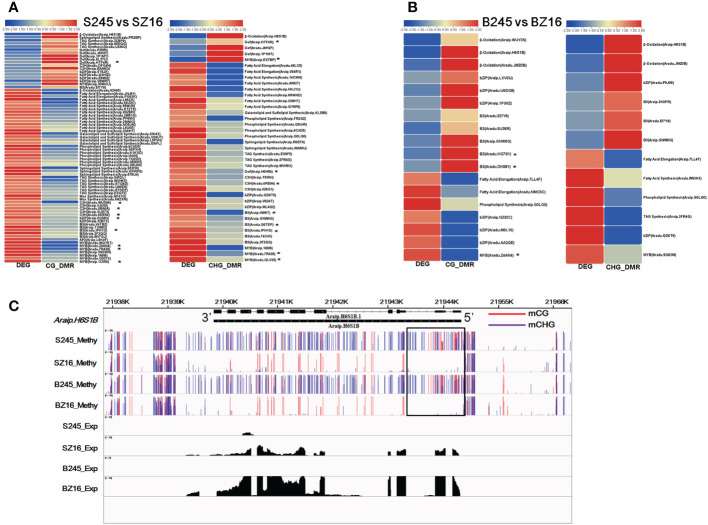
CG-DMR or CHG-DMR and their association with the expression of lipid metabolism-related genes. **(A, B)** Heatmaps showing CG-DMR and CHG-DMR between 245 and Z16 associated with differential expression at the R5 **(A)** and R8 **(B)** stages for sets of enzymes and TFs involved in lipid metabolism, respectively. * denote the CG-DMR- or CHG-DMR-associated TFs which were identified to co-express with lipid metabolic DEGs. Scales represent differential expression and differential methylation in log2 fold change. CG-DMR and CHG-DMR indicated differentially methylated regions in CG and CHG context, respectively. **(C)** A visualization showing the methylation level and expression level of acyl transporter (*Araip.H6S1B*) in seeds of 245 and Z16 at the R5 and R8 stages. S245 and SZ16 represented seeds of 245 and Z16 at the R5 stage, respectively. B245 and BZ16 denoted seeds of 245 and Z16 at the R8 stage, respectively.

There were 41 and 19 lipid metabolic DEGs that showed a negative correlation with DMRs in the CG and CHG contexts between 245 and Z16 at the R5 stage, respectively (**Figure 7A**). Most genes involved in FA synthesis, FA elongation, phospholipid synthesis, sphingolipid synthesis, galactolipid and sulfolipid synthesis, and TAG synthesis pathways exhibited hypomethylation with a higher expression in 245 at the R5 stage. In addition, 33 and 20 TFs known to be involved in the regulation of lipid metabolism were found to be negatively correlated with CG-DMR and CHG-DMR. Among the CG-DMR- or CHG-DMR-associated TFs, 15 members had been identified to co-express with lipid metabolic DEGs (**Figure 7A**, **Table S6**). Most TFs involved in the regulation of lipid metabolism, especially the co-expressed TFs, showed hypomethylation with higher abundance in 245 at the R5 stage. At the R8 stage, six and six lipid metabolic DEGs were found to be negatively correlated with CG-DMR and CHG-DMR between 245 and Z16, respectively (**Figure 7B**). Genes involved in β-oxidation showed hypermethylation with lower expression in 245 at the R8 stage. Reversely, genes involved in FA elongation, phospholipid synthesis, and TAG synthesis pathways showed hypermethylation with lower abundance in 245. Meanwhile, 17 TFs known to regulate lipid metabolism exhibited a negative correlation with DMR in the CG or CHG context at the R8 stage. Among them, three members were identified to co-express with lipid metabolic DEGs (**Figure 7B**, **Table S6**). Two DEGs showed hypermethylation with lower abundance, while another one showed hypomethylation with higher expression in 245 at the R8 stage. *Araip.H6S1B* encoding peroxisomal acyl transporter protein (*CTS*) was an example to exhibit a relationship between DMRs and lipid metabolic DEGs. The abundance of *CTS* was lower in 245 than that in Z16 at the two stages. Correspondingly, hyper-DMRs were observed at 5’ Untranslated Region (UTR) and upstream of *CTS* in 245 compared with Z16 (**Figure 7C**).

**Figure 8:**
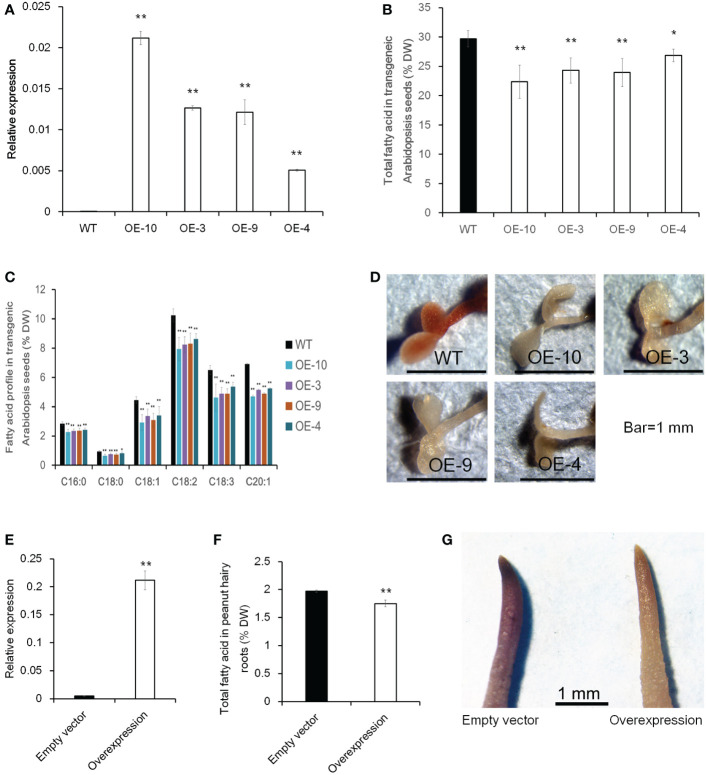
The effect of *CTS* overexpression on oil content in transgenic *Arabidopsis* and peanut hairy roots. **(A)** Expression level of *CTS* in Col-0 and transgenic *Arabidopsis* lines. Error bars indicated SD (n = 3). *AtACTIN7* (AT5G09810) was used as the internal reference gene. **(B)** Total fatty acid content of mature seeds in Col-0 and transgenic *Arabidopsis* plants. Error bars indicated SD (n = 4). **(C)** Fatty acid profile in Col-0 and transgenic *Arabidopsis* seeds. Error bars indicated SD (n = 4). The x-axis represented the species of fatty acid. **(D)** Fat red 7B staining of Col-0 and transgenic *Arabidopsis* plants. **(E)** Expression level of *CTS* in peanut transgenic hairy roots. Error bars indicated SD (n = 4). *GAPDH* (Morgante et al., 2011) was used as the internal reference gene. **(F)** Total fatty acid content in peanut transgenic hairy roots. Error bars indicated SD (n = 4). **(G)** Fat red 7B staining of peanut hairy roots. Student’s *t*-test was used for significance analysis. Asterisks indicated significant differences between transgenic plants with WT plants (empty vector) under the same treatment (* *P* < 0.05 and ** *P* < 0.01). *CTS* indicated acyl transporter (*Araip.H6S1B*).

To further investigate whether altering the expression level of *CTS* would influence oil accumulation, transgenic analysis in *Arabidopsis* and peanut hairy root was performed. The ORF of *CTS* was overexpressed using the CaMV 35S promoter in *Arabidopsis*, and 10 homozygous transgenic lines were obtained (**Figure S4**, **Figure 8A**). Then, four lines with a relatively higher expression level were selected to investigate total FA content in mature seed. In comparison with wild-type plants (Col-0), the overexpressing lines had a significantly lower total FA content and showed a decrease of 24.7% in OE-10, 18.3% in OE-3, 19.4% in OE-9, and 9.6% in OE-4 (**Figure 8B**). FA composition was also analyzed, and the examined FA species exhibited a significantly lower level in transgenic lines than those in Col-0 (**Figure 8C**). Five-day-old *Arabidopsis* seedlings were further stained with Sudan red 7B; less red color in overexpressing plants than Col-0 was observed (**Figure 8D**). In peanut, we used a transgenic hairy root system to test whether increasing *CTS* transcript abundance would decrease FA content in hairy roots. The expression level of *CTS* was much higher in overexpressing transgenic hairy roots than that in the control hairy roots (empty vector). Meanwhile, the total FA content of transgenic hairy roots was significantly lower than that of control roots (**Figures 8E, F** **)**. The stain assay also revealed a much lighter red color in overexpressing transgenic roots than in the roots containing empty vector (**Figure 8G**). These results indicated that overexpression of *CTS* would decrease oil content.

## Discussion

Cultivated peanut (*A. hypogaea*) was domesticated from the wild tetraploid *A. monticola*, originated by hybridization between two diploid *A. duranensis* and *A. ipaensis* (Bertioli et al., 2011; Moretzsohn et al., 2013; Yin et al., 2020). Compared to cultivated species, wild peanut possesses high genetic diversity and several superior features including a high oil content in seed (Huang et al., 2012). Understanding lipid accumulation in *A. monticola* at the level of regulation of gene expression would contribute to high-oil improvement in peanut breeding. In this study, we used the RNA-seq approach to analyze transcriptome divergence between wild and cultivated peanut at two seed developing stages (R5 and R8) and investigated the molecular mechanism underlying the difference in seed oil content. More than 70% of the reference genes (56,236) were identified and profiled in at least one sample. According to the result of PCA for expression pattern, the developmental stage prior to species was the first principal component to distinguish the samples. However, the expression profiles at the R8 stage could be obviously divided into two species in the hierarchical clustering diagram, indicating that gene expression profiles in the developing seed (R8 stage) have been altered during peanut domestication. Available literature suggests that gene expression divergence is essential to drive phenotypic variation during domestication (Lin et al., 2012; Koenig et al., 2013; Lu et al., 2016). Interestingly, we detected two “divergent” gene clusters (C2 and C7) in peanuts enriched in lipid metabolism (**Figure 2**). Our results suggest that there may be the existence of divergence at the gene expression level between wild and cultivated peanut contributing to the difference in lipid accumulation.

The lipid metabolism-related DEGs were presumed to play key roles in oil accumulation. Based on the expression pattern of lipid metabolic DEGs, samples could be clearly clustered into two groups (wild and cultivated) (**Figure 3**). Most DEGs in the lipid biosynthesis pathways were upregulated in wild species, especially at the lipid initial accumulation stage (R5) (**Figure 3**). Meanwhile, β-oxidation-related DEGs, which are involved in the degradation of lipid, were downregulated in wild peanut from lipid initiation to lipid rapid increase stages (R5 and R8). The DEG analysis revealed a robust activity of oil biosynthesis with a constraint of lipid degradation in developing seed of wild peanuts. It is worth noting that 16 lipid metabolic DEGs between wild and cultivated peanut were repeatedly identified at both R5 and R8 stages. These consistently divergent genes during seed development may contribute to a part of the difference in oil accumulation between wild and cultivated species. For example, *Aradu.CP1HR* encoded a biotin carboxyl carrier protein, which is a subunit of acetyl-CoA carboxylase (ACC). ACC catalyzes malonyl-CoA and bicarbonate to yield malonyl-CoA, which is the first committed step in FA synthesis (Li-Beisson, 2013). The expression level of *Aradu.CP1HR* was upregulated in wild species. In contrast, the expression abundance of another gene (*Araip.H6S1B*) was lower in wild species. It was an acyl transporter protein involved in the import of β-oxidation substrate (FAs) to peroxisome to break down FAs (Li-Beisson, 2013). In addition, many TFs known in the regulatory circuitry during seed development (Agarwal et al., 2011; Li and Li, 2016) were identified to co-express with lipid metabolic DEGs (**Figure 4**). The TF families, such as AP2, B3, b-ZIP, and C3H, whose members were grouped into “divergent” gene cluster C2 with different abundance between wild and cultivated peanuts (**Figure 4**, **Figure S2**, **Table S6**), have been well known in the regulation of oil production (Pouvreau et al., 2011; Song et al., 2013; Kim et al., 2015; Manan et al., 2017; Lu et al., 2021). Meanwhile, approximately 44% of lipid metabolic-related DEGs were also categorized into gene cluster C2. Since a set of genes with a similar expression pattern was likely to take part in the same biological process, we deduced that the co-expressed network of TFs and lipid metabolic DEGs may construct transcriptional modules affecting differential oil accumulation between wild and cultivated peanuts. Altogether, TFs may coordinate the expression abundance of lipid metabolic genes to promote oil biosynthesis in wild species. It might explain the higher oil content in wild peanuts than in cultivated peanuts.

**Figure 9:**
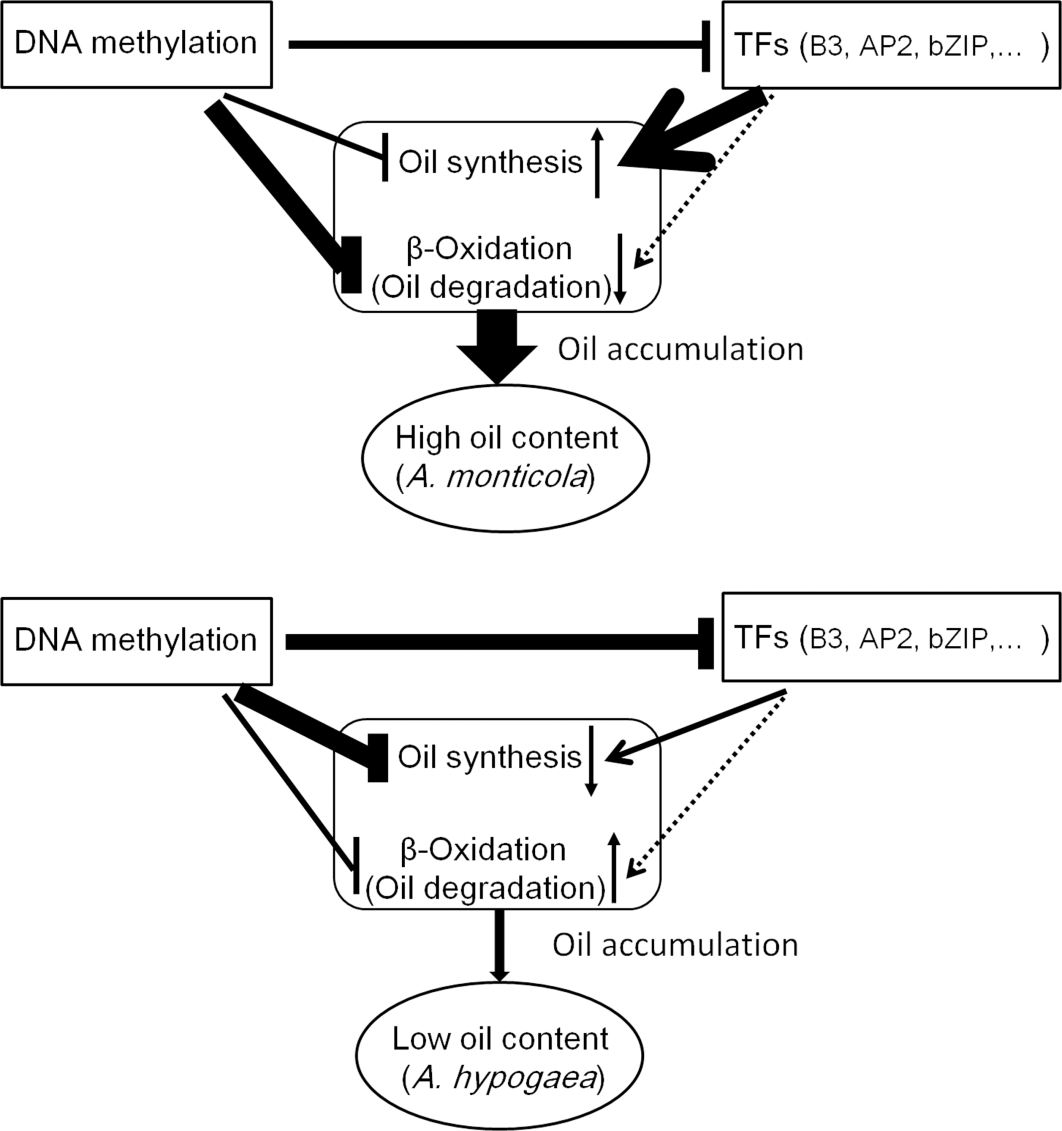
A regulatory model of lipid accumulation in peanut seed. Compared to *A. hypogaea*, *A. monticola* activated the transcription of genes involved in oil synthesis and depressed the transcription of genes involved in β-oxidation *via* DNA methylation and TFs in seed. Thus, DNA methylation and TFs coordinately tuning the expression of genes contribute to high oil accumulation in *A. monticola*.

DNA methylation is a conserved epigenetic marker that regulates gene expression. There are examples of natural epialleles in several crops showing varied DNA methylation affecting multiple biological processes (Manning et al., 2006; Zhang et al., 2015; Song et al., 2017). Here, we sought to investigate the role of DNA methylation on genes involved in lipid accumulation. The genome-wide DNA methylome and trancriptome for developing seeds of 245 (wild species) and Z16 (cultivated species) were conjointly profiled (**Figure 5**). A significantly negative correlation on chromosome-scale between DNA methylation and gene expression was observed, indicating that DNA methylation generally inhibits gene transcription in developing peanut seeds (R5 and R8). Meanwhile, DMRs between 245 and Z16 were displayed on 20 reference chromosomes (**Figures 6A, B**), providing a first glimpse of the epigenetic changes in seed development during peanut domestication. DMRs in CG and CHG, but not in the CHH context, were positively correlated with gene density on chromosomes and tended to enrich in genic regions (gene body+2k upstream) of DEGs (**Figure 6D**). Previous studies have shown that DNA methylation occurring in the promoter or within the transcribed gene body would regulate gene transcription (Zhang et al., 2006; Wang et al., 2015; Bewick and Schmitz, 2017; Lang et al., 2017; Huang et al., 2019). Therefore, the DMRs, especially in CG and CHG contexts, would play a role in the differential expression of genes in the present study. There were 50% and 25% of DEGs between 245 and Z16 that were associated with CG-DMRs and CHG-DMRs, respectively. GO terms of CG-DMR- and CHG-DMR-associated DEGs were enriched in several biological processes, including lipid metabolism. There was an obvious trend that most differentially expressed enzymes and co-expressed TFs involved in lipid production were hypomethylated in wild peanut (245) at the R5 stage. In contrast, DEGs involved in β-oxidation were found hypermethylated in 245 at the R8 stage. Acyl transporter protein (*CTS*, *Araip.H6S1B*) that takes part in the β-oxidation process to degrade lipid was an example to demonstrate that the influence of DNA methylation on the expression of lipid-related genes varied between wild (245) and cultivated peanut (Z16, **Figure 7C**). Hypomethylated promoter and 5’ UTR of *CTS* (*Araip.H6S1B*) with increased expression abundance were observed in low-oil content peanut (Z16), and the transgenic assay further confirmed that enhancing the expression of *CTS* (*Araip.H6S1B*) would reduce oil content in *Arabidopsis* seeds and peanut hairy root (**Figure 8**). It was suggested that DNA methylation may play an important role in oil accumulation through regulating the expression of specific genes including *CTS*. In addition, 20 TFs including AP2 (*Aradu.C87QH*) were predicted to bind the promoter (2 kb upstream) of the *CTS* gene (**Figure S5**). The abundance of most putative binding TFs was relatively higher in wild peanut (245) than that in cultivated peanut (Z16). In contrast, their target gene (*CTS*) was expressed more in cultivated peanut (Z16), suggesting that AP2 and other TFs may bind promote *CTS* to suppress its transcription. Altogether, the methylome and transcriptome data depicted a possible regulatory network in which DNA methylation and TFs coregulate the expression of lipid metabolism genes in peanut seed (**Figure 9**). In wild peanut, comprehensive alteration of gene transcription by TFs and DNA methylation would promote oil production and constrain oil degradation simultaneously, which finally contribute to higher oil accumulation.

In summary, transcriptomic and methylomic comparisons revealed gene expression and DNA methylation variations in seed development between wild and cultivated peanuts. In wild peanut seed, TFs and DNA methylation may coordinately regulate specific lipid metabolic genes to activate oil biosynthesis and simultaneously constrain lipid degradation. Thus, gene expression change would contribute to increasing oil content in wild peanut. Our study reveals a regulation mechanism of oil accumulation in seed and provides gene resources for oil improvement in cultivated peanut.

## Data availability statement

The datasets presented in this study can be found in online repositories. The names of the repository/repositories and accession number(s) can be found below: https://www.ncbi.nlm.nih.gov/, PRJNA781013, https://www.ncbi.nlm.nih.gov/, PRJNA782686.

## Author contributions

NL, MP, RV, and HJ conceived, designed, and supervised the experiments. LH, YC, XZ, and JG managed materials planted in the experimental nursery. NL, BW, HL, WC, and DH conducted sampling and phenotyping. NL, BW, and BY performed DNA and RNA extraction. NL and BW performed bioinformatic analysis and interpreted the results. NL and BW prepared the first draft, and NL, MP, HC, YL, BL, RV, and HJ contributed to the final editing of manuscript. All authors read and approved the final manuscript.
