# Supplementary material for: Gene expression and DNA methylation altering lead to the high oil content in wild allotetraploid peanut (A. monticola)
Source: Front Plant Sci. 2022 Dec 16;13:1065267. doi: 10.3389/fpls.2022.1065267 (PMC9802669; doi:10.3389/fpls.2022.1065267)
Supplement: Supplementary file 1 [file DataSheet_1.pdf]

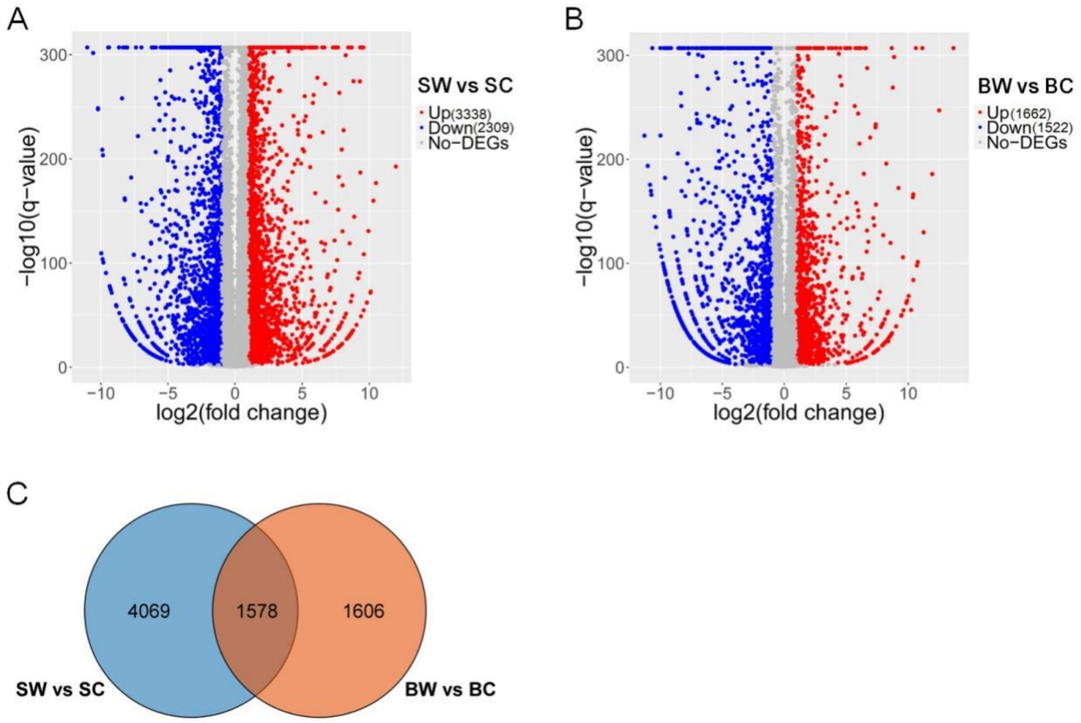

Figure S1. Differentially expressed genes (DEGs) between wild and cultivated peanuts

(A and B) Volcano plots of Up- and Down-regulated DEGs at R5 (A) and R8 (B) stages, respectively. (C) A venn diagram of the number of DEGs between wild and cultivated peanuts at R5 and R8 stages. SW and SC indicated seeds of wild and .cultivated peanuts at R5 stage, respectively. BW and BC denoted seeds of wild and cultivated peanuts at R8 stage, respectively.

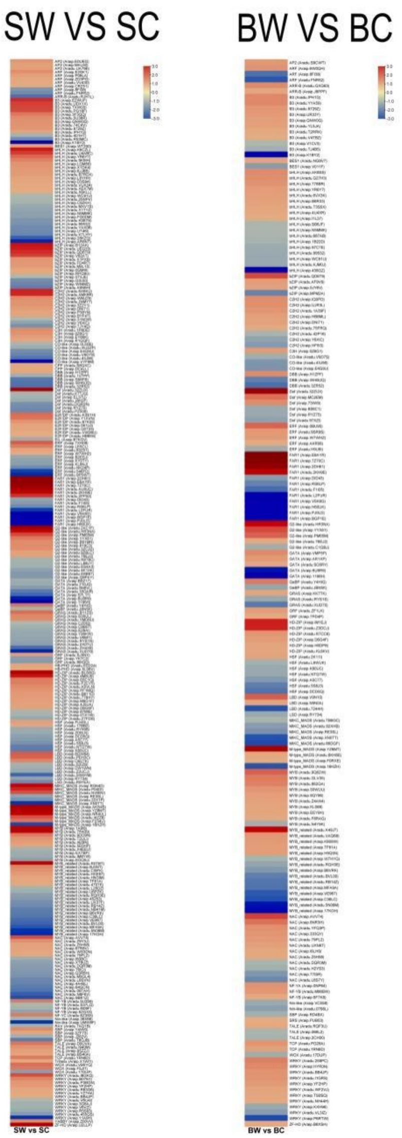

Figure S2. Heatmap of lipid metabolism-related DEGs between wild and cultivated peanuts

SW and SC indicated seeds of wild and cultivated peanuts at R5 stage, respectively. BW and BC denoted seeds of wild and cultivated peanuts at R8 stage, respectively.

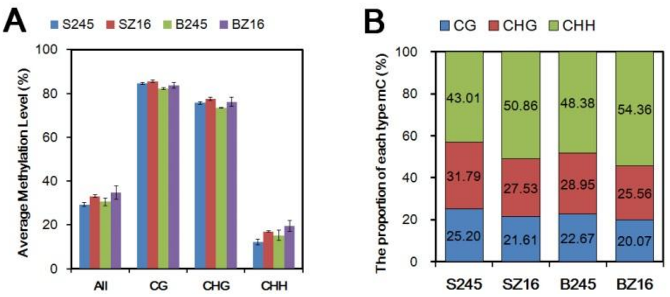

Figure S3. Global information of DNA methylation in seeds of S245 and Z16 at R5 and R8 stages

(A) The average methylation level of overall, CG, CHG, and CHH contexts. (B) The proportion of mCG, mCHG, mCHH among all methylcytosine. S245 and SZ16 represented seeds of 245 and Z16 at R5 stage, respectively. B245 and BZ16 represented seeds of 245 and Z16 at R8 stage, respectively.

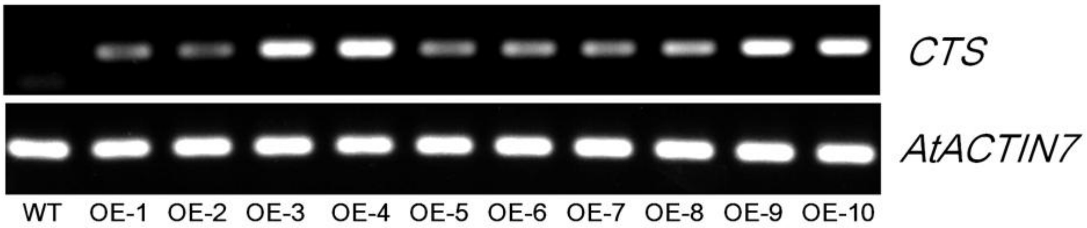

Figure S4. RT-PCR analysis of *CTS* expression in Col-0 and transgenic Arabidopsis plants.

The relative expression levels calculated using *AtACTIN7* (AT5G09810) as a reference gene. WT denoted Col-0, OE-1 to OE-10 represented transgenic Arabidopsis lines.

A

Binding Protein	TF Annotation	Log2(S245/SZ16)	Log2(B245/BZ16)
Aradu.C87QH	AP2	1.02	1.56
Aradu.MF3XQ	ARF	1.39	1.06
Aradu.YC3RY	ARF	0.14	-1.20
Araip.E2SK1	ARF	1.99	0.33
Araip.E51AM	ARF	1.76	1.29
Aradu.0TU0Y	B3	-0.17	-0.17
Araip.VT864	B3	0.14	0.24
Aradu.0K64L	BBR-BPC	0.64	0.15
Araip.86VYR	BBR-BPC	0.98	0.40
Araip.98LTB	BBR-BPC	0.31	-0.40
Aradu.898CQ	C2H2	0.20	0.38
Araip.M9JCH	C2H2	0.32	0.20
Araip.W28KY	Dof	-0.52	1.24
Aradu.9SR9S	ERF	0.49	2.36
Araip.KHB2Z	MYB	-0.05	#N/A
Aradu.7FN1T	Trihelix	3.02	#N/A
Aradu.84KC0	Trihelix	0.26	0.84
Araip.H9ZRK	Trihelix	0.47	0.10
Araip.X1WI1	Trihelix	1.87	#N/A
Araip.Z39NZ	Trihelix	0.53	0.44

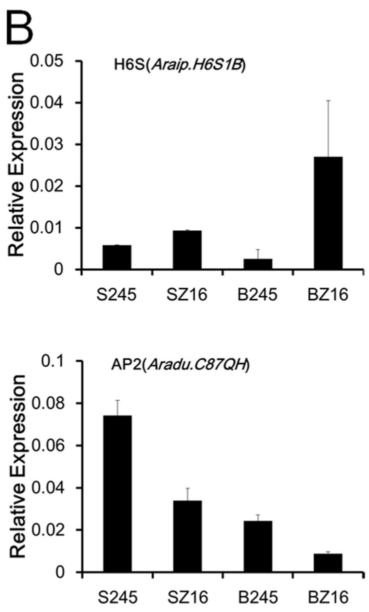

Figure S5. Analysis of putative transcription factors binding to *CTS.*
(A) List of putative binding TFs and their expression fold change between wild and cultivated peants. Prediction of TFs is performed on the website PlantRegMap (http://plantregmap.gao-lab.org/binding_site_prediction.php). (B) Divergent expression of AP2 (*Aradu.C87QH*) and its target (*CTS*). Error bars indicated SD (n=3). *GAPDH* (Morgante et al., 2011) was used as the internal reference gene.
